# The EVIDENT diet quality index is associated with cardiovascular risk and arterial stiffness in adults

**DOI:** 10.1186/s12889-017-4194-y

**Published:** 2017-04-08

**Authors:** Carmela Rodríguez-Martin, Rosario Alonso-Domínguez, María C Patino-Alonso, Manuel A Gómez-Marcos, José A Maderuelo-Fernández, Carlos Martin-Cantera, Luis García-Ortiz, José I. Recio-Rodríguez, on behalf of the EVIDENT group, L. García Ortiz, M. A. Gómez Marcos, J. I. Recio Rodríguez, M. C. Castaño Sánchez, C. Rodríguez Martin, C. Agudo Conde, E. Rodríguez Sánchez, L. J. González Elena, C. Herrero Rodríguez, B. Sánchez Salgado, A. de Cabo Laso, J. A. Maderuelo Fernández, C. Martin Cantera, J. Canales Reina, E. Rodrigo de Pablo, M. L. Lasaosa Medina, M. J. Calvo Aponte, A. Rodríguez Franco, E. Briones Carrio, C. Martin Borras, A. Puig Ribera, R. Colominas Garrido, J. Anton Álvarez, Ma. T. Vidal Sarmiento, A. Viaplana Serra, S. Bermúdez Chillida, A. Tanasa, M. Romaguera Bosch, M. M. Domingo, A. Girona, N. Curos, F. J. Mezquiriz, L. Torrent, A. Cabrejas Sánchez, M. T. Pérez Rodríguez, M. L. García García, J. L. Bartolomé, F. Salcedo Aguilar, C. Fernández Alonso, A. Gómez Arranz, E. Ibáñez Jalón, A. de la Cal de la Fuente, N. Gutiérrez, L. Muñoz, M. Menéndez, I. Repiso, R. Sanz Cantalapiedra, L. M. Quintero González, S. de Francisco Velasco, M. A. Diez García, E. Sierra Quintana, M. Caceres, N. González Viejo, J. F. Magdalena Belio, L. Otegui Ilarduya, F. Ja. Rubio Galán, A. Melguizo Bejar, C. I. Sauras Yera, Ma. J. Gil Train, M. Iribarne Ferrer, M. A. Lafuente Ripolles, G. Grandes, A. Sánchez, N. Guenaga, V. Arce, M. S. Arietaleanizbeaskoa, E. Iturregui San Nicolás, R. A. Martín Santidrian, A. Zuazagoitia

**Affiliations:** 1 Primary Care Research Unit, The Alamedilla Health Center, Castilla and León Health Service (SACYL), Biomedical Research Institute of Salamanca (IBSAL), Spanish Network for Preventive Activities and Health Promotion (redIAPP), Salamanca, Spain; 2 Department of statistics, University of Salamanca, Biomedical Research Institute of Salamanca (IBSAL), Spanish Network for Preventive Activities and Health Promotion (redIAPP), Salamanca, Spain; 3 Primary Care Research Unit, The Alamedilla Health Center, Castilla and León Health Service (SACYL), Biomedical Research Institute of Salamanca (IBSAL), Department of medicine, University of Salamanca, Spanish Network for Preventive Activities and Health Promotion (redIAPP), Salamanca, Spain; 4 Passeig de Sant Joan Health Center, Catalan Health Service, Spanish Network for Preventive Activities and Health Promotion (redIAPP), Barcelona, Spain; 5 Primary Care Research Unit, The Alamedilla Health Center, Castilla and León Health Service (SACYL), Biomedical Research Institute of Salamanca (IBSAL), Department of biomedical and diagnostic sciences, University of Salamanca, Spanish Network for Preventive Activities and Health Promotion (redIAPP), Salamanca, Spain; 6 Primary Care Research Unit, The Alamedilla Health Center, Castilla and León Health Service (SACYL), Biomedical Research Institute of Salamanca (IBSAL), Spanish Network for Preventive Activities and Health Promotion (redIAPP), Department of Nursing and Physiotherapy, University of Salamanca, Avda. Comuneros N° 27, 37003 Salamanca, Spain

**Keywords:** Food habits, Risk factors, Vascular stiffness, Diet, Mediterranean

## Abstract

**Background:**

We aimed to simplify information from food frequency questionnaires (FFQs) in a single parameter that allows for rapid identification of quality of patient diet and its relationship to cardiovascular risk and pulse wave velocity (PWV).

**Methods:**

The sample from the EVIDENT study, consisting of 1553 subjects (aged 20–80 years) with no cardiovascular disease selected by random sampling among those attending primary care clinics, was used. The EVIDENT diet index (range 0–100) was calculated based on the results of a FFQ. Evaluation of dietary habits also included adherence to the Mediterranean diet (MD). Cardiovascular risk was estimated, and carotid-femoral pulse wave velocity was measured.

**Results:**

Mean subject age was 54.9 ± 13.8 years, and 60.3% of subjects were female. The mean value of the EVIDENT diet index was 52.1 ± 3.2 points. Subjects in the third tertile (the highest score) had the greatest adherence to MD and the highest energy intake, with greater amounts of carbohydrates, protein, and fiber. The best cut-off point of the EVIDENT diet index for predicting good adherence to the MD is 52.3 (0.71 sensitivity, 0.61 specificity). In a multiple regression analysis, after a complete adjustment, it was estimated that for each one-point increase in the EVIDENT diet index, cardiovascular risk (CVR), blood-pressure, waist circumference, and PWV decreased by 0.14, 0.43, 0.24, and 0.09 respectively (*p* < 0.05, all).

**Conclusions:**

The diet quality index developed is associated to CVR and its components, and also with arterial stiffness, as measured with PWV. This index is also a good predictor of adherence to MD.

## Background

Lifestyle has been shown to be an essential determinant for the presence or absence of many cardiovascular risk factors. More specifically, wide scientific evidence exists of the relationship between dietary habits and development of cardiovascular diseases [1, 2].

Epidemiological studies aimed at analyzing the relationship between diet and chronic disease use different tools to collect information on the usual diet during a given period of time. Food frequency questionnaires (FFQs) are among the most commonly used methods [3]. In Spain, the FFQ developed and used in the PREDIMED study [4] has often been used and has provided ample evidence of the benefits of the Mediterranean diet for cardiovascular health [2]. However, routine use of this FFQ in daily practice is unfeasible because of its complexity, and this questionnaire has mainly been relegated to the field of research. Recently, questionnaires that evaluate the quality of the diet have been developed, adapted to different population contexts [5–8].

The purpose of this study was to evaluate the relationship of a diet quality index derived from a food frequency questionnaire with the cardiovascular risk and pulse wave velocity in a sample of Spanish adults. As a secondary objective we want to evaluate how this index predicts adherence to the Mediterranean diet.

## Methods

### Study design

The results of this study are a subanalysis of the EVIDENT study (from the Spanish title, Estilos de vida y disfunción endotelial), which was intended to evaluate the relationship of lifestyle with circadian blood pressure pattern, arterial stiffness, and endothelial function in a cohort of adults with no cardiovascular disease. This was a cross-sectional multicenter study with the participation of six Spanish primary care centers. The protocol of the EVIDENT study [9], describing the methods used to collect information, has previously been reported.

### Study population

One thousand five hundred fifty three subjects aged 20 to 80 years were selected by systematic random sampling in primary care clinics. Exclusion criteria were: coronary or cerebrovascular atherosclerotic disease, heart failure, moderate to severe chronic obstructive pulmonary disease, musculoskeletal disease that prevented walking, advanced liver, lung or kidney disease, severe mental disease, oncological disease treated and diagnosed within five years of study start, end-stage disease, and pregnancy.

### Variables and measurement instruments

#### Sociodemographic and lifestyle variables

Age, sex, occupation, smoking (non smokers, exsmokers and current smokers), and alcohol consumption were evaluated by clinical interview.

#### Clinical history

Cardiovascular risk factors (high blood pressure, dyslipidemia, and diabetes) and use of drugs were evaluated through the electronic medical record consultation, physical examination and clinical interview.

Risk of cardiovascular morbidity and mortality was estimated using the published risk equation (D’Agostino scale) based on the Framingham study [10]. Risk factors for morbidity and mortality used by the Framingham Risk Score include age, sex, total cholesterol, high-density lipoprotein cholesterol, and SBP as quantitative variables, and drug treatment for hypertension, smoking, and history of diabetes mellitus as dichotomous variables.

#### Blood pressure measurement

Systolic (SBP) and diastolic blood pressure (DBP) were measured three times in each arm, and the mean of the last two measurements in the arm with higher values was used. Measurements were performed with an OMRON model M7 blood pressure monitor (Omron Health Care, Kyoto, Japan) following the recommendations of the European Society of Hypertension [11].

#### Anthropometric variables

Body weight was measured twice with a certified electronic balance (Seca 770) after adequate calibration (precision ±0.1 kg). Readings were rounded to 100 g. Height was measured with a stadiometer (Seca 222), recording the mean of two measurements. Body mass index (BMI) was calculated using the formula: weight in kg divided by height in meters squared considering obesity a BMI ≥ 30 Kg/m^2^. Waist circumference (WC) was measured with a flexible measuring tape. All anthropometric measurements were evaluated following the 2007 recommendations of the Spanish Society for the Study of Obesity [12]. All measurements were made with the patient standing, with no shoes and wearing light clothing.

#### Analysis of pulse wave velocity (PWV)

The Salamanca cohort (Group of participants recruited in Salamanca *N* = 263) was measured the pulse wave velocity. Measurement was performed with the subject in supine position and using the Sphygmocor System (AtCor Medical Pty Ltd. Head Office, West Ryde, Australia), following the consensus recommendations of Van Bortel et al. [13]. Pulse wave in the carotid and femoral arteries was analyzed, and delay with regard to ECG wave was estimated. Distance measurements were taken with a measuring tape from the sternal notch to the point where sensor was located.

#### Diet

Dietary intake was recorded using a self-administered, semiquantitative FFQ. This questionnaire has been validated for Spain [3] and includes 137 food items frequently used by the reference population. After receiving instructions from the study staff, participants have indicated the frequency each food item had been consumed in the past year using a 9-item scale (never or almost never, 1–3 times monthly, once weekly, 2–4 times weekly, 5–6 times weekly, once daily, 2–3 times daily, 4–6 times daily, or more than 6 times daily). This questionnaire allows for estimating daily intake of energy, immediate principles, and others. The EVIDENT diet index was created based on the FFQ including the 137 foods that are collected in this questionnaire. This index scores each item depending on whether the food is considered positive, negative, or neutral for health. This consideration was based on the dietary patterns proposed in the study of Nettleton et al. [14] adapted to the Spanish population dietary habits. As consumption increases, score of food items considered positive increases by between 0 and 8 points, while score of those considered negative decreases by between 8 and 0 points. Neutral foods are not scored. This results in a global score which, for better understanding, has been standardized to a 0–100 point range. Higher scores are considered representative of better quality of diet. Table 1 provides more details of calculation of this index. Adherence to Mediterranean diet (MD) was also assessed using the 14-item validated questionnaire (MEDAS) [5]. This questionnaire includes 14 items with two possible answers. Each answer considered positive for health counted as one point. The final score ranged from 0 and 14 points. Adherence to Mediterranean diet was considered good when score was 9 points or higher [5].

**Table 1:** Composition of the EVIDENT diet index

Positive food groups			
Never or almost never	0 points	Low-fat dairy (milk, yogurt, cheese)	Boiled or roasted potatoes
1–3 times per month	1 point	Poultry	Fruits
Once per week	2 points	Rabbit	Fresh fruit juice
2–4 times per week	3 points	Fish	Beans, lentils, chickpeas
5–6 times per week	4 points	Dark-yellow vegetables	Whole-grain bread, rice, cereal or pasta
Once per day	5 points	Green leafy vegetables	Olive oil
2–3 times per day	6 points	Cruciferous vegetables	Green or black tea
4–6 times per day	7 points	Other vegetables	Red wine
More than six times per day	8 points	Gazpacho^a^	Beer
Negative food groups			
Never or almost never	8 points	Whole-fat dairy (milk, yogurt, cheese)	Sweet breads
1–3 times per month	7 points	Ice cream	Desserts
Once per week	6 points	Red meat	Added sweets
2–4 times per week	5 points	Processed meat	Pre-cooked meals
5–6 times per week	4 points	Pizza	Sauce (ketchup, mayonnaise)
Once per day	3 points	Fried potatoes	Honey
2–3 times per day	2 points	Salty snack foods	Jam
4–6 times per day	1 point	Added fats and oils	Soda
More than six times per day	0 points	Butter	Bottled juices
Neutral food groups			
		Semi-skimmed milk	Whole grain biscuits
		Eggs	Chocolate
		Jamón Serrano^b^	Diet soda
		Refined-grain bread, rice, cereal or pasta	Other alcohol

^a^Cold vegetable soup

^b^Type of top-quality Parma ham

#### Laboratory variables

A blood sample was collected after a 12-h fast for measuring lipids, glucose, HbA1c and insulin. Serum total cholesterol, HDL-cholesterol and triglyceride concentrations were measured using standard automated enzymatic methods. LDL cholesterol was estimated using the Friedewald equation when the direct parameter was not available.

### Statistical analysis

Results are given as mean ± standard deviation for quantitative variables or as frequency distributions for qualitative variables. Differences in means of continuous variables between tertiles (T) of EVIDENT diet index (with T1 being the lowest and T3 the highest) were analyzed through a one-way analysis of variance (ANOVA) for independent samples, using the Fisher’s Least Significant Difference (*LSD*) method in post hoc contrasts. A multiple linear regression analysis was performed, including the EVIDENT index as independent variable and cardiovascular risk, BP, waist circumference, and PWV as dependent variables. A first unadjusted model and a second model adjusted by age and sex were performed. Finally, a third model adjusted for age, sex, smoking, energy intake, SBP, and antihypertensive, antidiabetic, and lipid-lowering treatment was used. ROC curve analysis was performed to assess sensitivity, specificity, and area under the curve of the EVIDENT diet index to determine the optimum cut-off point defining good adherence to Mediterranean diet in relation to MEDAS. Statistical analysis was performed using IBM SPSS Statistics for Windows, Version 23.0. Armonk, NY: IBM Corp. A value of *p* < 0.05 was considered statistically significant.

## Results

A total of 1553 subjects with a mean age of 54.9 ± 13.8 years were enrolled into the study. Of these, 60.3% were females. The mean value of the EVIDENT diet index was 52.1 ± 3.2 points. The baseline characteristics by tertiles of EVIDENT index score were similar in both sexes. T1 (the tertile with the lowest score, <50.1 for men and <51.4 for women) had the lowest mean age, a smaller proportion of subjects with dyslipidemia or on lipid-lowering drugs, and a lower estimated cardiovascular risk. By contrast, T3 (the tertile with the highest score, >52.9 for men and <53.8 for women) was the oldest. Tables 2 and 3.

**Table 2:** Baseline characteristics by tertiles of EVIDENT index score (Men (*n* = 616))

	TERTILE 1<50.12	TERTILE 2 (50.12 to 52.98)	TERTILE 3 >52.98	
	Mean or N (SD or %)	Mean or N (SD or %)	Mean or N (SD or %)	*p* value
Age (years)	50.4 ± 14.1	58.5 ± 13.1	60.9 ± 11.4	<0.001
Hypertension (n, %)	85 (45.9)	111 (56.9)	107 (58.5)	0.031
Diabetes (n, %)	19 (10.2)	35 (17.9)	34 (18.4)	0.051
Dyslipidemia (n, %)	61 (34.3)	92 (47.2)	107 (58.2)	<0.001
Antihypertensive agents (n, %)	52 (28.0)	68 (34.7)	83 (44.9)	0.003
Antidiabetic agents (n, %)	15 (8.1)	23 (11.7)	28 (15.1)	0.105
Lipid-lowering agents (n, %)	26 (14.0)	58 (29.6)	63 (34.1)	<0.001
SBP (mmHg)	129.4 ± 15.3	132.2 ± 15.0	129.9 ± 14.0	0.155
DBP (mmHg)	79.4 ± 11.1	79.3 ± 9.4	78.7 ± 9.5	0.735
Heart rate (bpm)	70.5 ± 11.7	71.3 ± 11.4	68.6 ± 12.9	0.090
Insulinemia (mg/dL)	8.81 ± 6.99	8.89 ± 5.38	7.49 ± 5.19	0.049
Glycosilated haemoglobin (%)	5.60 ± 0.83	5.74 ± 0.67	5.81 ± 0.85	0.039
Glucose (mg/dL)	94.3 ± 27.0	97.1 ± 19.3	101.0 ± 28.0	0.037
Total cholesterol (mg/dL)	203.3 ± 40.7	210.2 ± 37.9	212.3 ± 38.0	0.070
Triglycerides (mg/dL)	147.0 ± 148.8	130.4 ± 84.1	142.6 ± 158.4	0.463
HDL-cholesterol (mg/dL)	50.1 ± 12.0	51.7 ± 12.0	54.0 ± 13.1	0.012
LDL-cholesterol (mg/dL)	131.3 (45.0)	133.1 (32.6)	134.6 (32.8)	0.701
BMI (Kg/m^2^)	27.9 ± 4.2	28.1 ± 3.5	27.3 ± 3.9	0.076
Obesity (n, %)	49 (26.3)	56 (28.6)	32 (17.3)	0.026
Waist circumference (cm)	98.5 ± 11.4	99.7 ± 9.4	96.8 ± 11.4	0.031
Smoking status				
Non smoker (n, %)	61 (32.8)	66 (33.7)	65 (35.1)	<0.001
Exsmoker (n, %)	63 (33.9)	96 (49.0)	94 (50.8)	
Current smoker (n, %)	62 (33.3)	34 (17.3)	26 (14.1)	
RCV (D’Agostino)	16.3 ± 13.2	22.6 ± 15.5	23.9 ± 17.0	<0.001
PWV (m/s) (*n* = 263)	8.13 ± 1.95	8.99 ± 2.93	7.84 ± 2.02	0.158

Categorical variables are expressed as n (%) and continuous variables as mean ± standard deviation

p: statistically significant differences (*p* < 0.05)

*T* Tertile, *SBP* Systolic blood pressure, *DBP* Diastolic blood pressure, *BMI* Body mass index, *CVR* Cardiovascular risk, *PWV* Pulse wave velocity

**Table 3:** Baseline characteristics by tertiles of EVIDENT index score (Women (*n* = 937))

	TERTILE 1 <51.43	TERTILE 2 (51.43 to 53.85)	TERTILE 3 >53.85	
	Mean or N (SD or %)	Mean or N (SD or %)	Mean or N (SD or %)	*p* value
Age (years)	48.8 ± 14.6	55.1 ± 13.5	57.6 ± 11.5	<0.001
Hypertension (n, %)	87 (29.4)	112 (38.5)	105 (35.8)	0.058
Diabetes (n, %)	22 (7.4)	23 (7.9)	27 (9.1)	0.749
Dyslipidemia (n, %)	94 (33.2)	129 (45.3)	134 (45.9)	0.003
Antihypertensive agents (n, %)	61 (20.6)	75 (25.8)	78 (26.3)	0.206
Antidiabetic agents (n, %)	11 (3.7)	17 (5.8)	21 (7.1)	0.196
Lipid-lowering agents (n, %)	25 (8.4)	42 (14.4)	64 (21.5)	<0.001
SBP (mmHg)	119.8 ± 16.9	122.2 ± 18.2	120.4 ± 17.0	0.226
DBP (mmHg)	74.9 ± 11.1	76.0 ± 10.3	75.7 ± 10.3	0.478
Heart rate (bpm)	75.2 ± 11.4	72.1 ± 10.5	72.2 ± 10.4	<0.001
Insulinemia (mg/dL)	8.10 ± 5.92	7.44 ± 6.02	7.72 ± 5.29	0.420
Glycosilated haemoglobin (%)	5.51 ± 0.72	5.64 ± 0.70	5.63 ± 0.72	0.058
Glucose (mg/dL)	90.8 ± 22.6	92.2 ± 18.8	93.0 ± 22.4	0.453
Total cholesterol (mg/dL)	212.7 ± 38.9	221.3 ± 39.4	215.9 ± 35.9	0.025
Triglycerides (mg/dL)	102.4 ± 58.9	110.4 ± 90.8	113.4 ± 113.4	0.329
HDL-cholesterol (mg/dL)	62.7 ± 16.6	64.1 ± 15.3	63.4 ± 14.7	0.555
LDL-cholesterol (mg/dL)	129.5 ± 34.4	136.5 ± 35.8	132.3 ± 32.6	0.050
BMI (Kg/m^2^)	26.2 ± 5.1	26.9 ± 5.2	26.3 ± 4.2	0.204
Obesity (n, %)	61 (20.6)	65 (22.3)	44 (14.9)	0.058
Waist circumference (cm)	88.7 ± 12.8	90.5 ± 11.5	89.6 ± 11.1	0.166
Smoking status				
Non smoker (n, %)	146 (49.5)	169 (58.1)	186 (62.6)	<0.001
Exsmoker (n, %)	58 (19.7)	67 (23.0)	64 (21.6)	
Current smoker (n, %)	91 (30.8)	55 (18.9)	47 (15.8)	
RCV (D’Agostino)	7.1 ± 7.6	9.0 ± 9.9	8.5 ± 7.2	0.020
PWV (m/s) (*n* = 263)	6.98 ± 1.67	7.46 ± 1.73	7.12 ± 1.73	0.386

Categorical variables are expressed as n (%) and continuous variables as mean ± standard deviation

p: statistically significant differences (*p* < 0.05)

*T* Tertile, *SBP* Systolic blood pressure, *DBP* Diastolic blood pressure, *BMI* Body mass index, *CVR* Cardiovascular risk, *PWV* Pulse wave velocity

In both sexes, T3 had the highest energy intake, with greater amounts of carbohydrates, protein, and fiber and lower consumption of cholesterol and saturated fat-except for energy intake in men. By contrast, subjects in T1 had the greatest intake of fat (saturated fat and cholesterol) and lower consumption of fiber. Differences were found in adherence to Mediterranean diet evaluated, with the lowest and highest scores in MEDAS being found in T1 and T3 respectively (6.4 ± 1.7 vs. 8.6 ± 1.8, *p* < 0.001 in men; 6.8 ± 1.9 vs. 8.6 ± 1.6, *p* < 0.001 in women). Tables 4 and 5.

**Table 4:** Energy intake and daily nutrients intake by tertiles of the EVIDENT diet index (Men (*n* = 616))

	TERTILE 1 <50.12	TERTILE 2 (50.12 to 52.98)	TERTILE 3 > 52.98	
	Mean ± SD	Mean ± SD	Mean ± SD	*p* value
Energy intake(Kcal/day)^a^	2571.6 ± 902.6	2353.1 ± 735.0	2531.4 ± 1051.2	0.042
Carbohydrate (g/day)^c^	271.0 ± 119.4	249.6 ± 93.6	284.8 ± 189.5	0.046
Protein (g/day)^c^	103.0 ± 32.3	99.8 ± 28.7	109.3 ± 45.4	0.033
Total fat (g/day)^ab^	108.5 ± 41.8	92.8 ± 32.9	92.8 ± 30.3	<0.001
Saturated fat (g/day)^ab^	35.7 ± 13.0	28.1 ± 10.3	25.9 ± 9.3	<0.001
Fiber (g/day)^abc^	18.9 ± 9.1	23.1 ± 8.0	33.1 ± 28.5	<0.001
Cholesterol (g/day)^ab^	512.8 ± 222.4	461.2 ± 159.4	448.2 ± 177.8	0.002
Carbohydrate (%)^bc^	41.55 ± 7.39	42.00 ± 6.99	44.11 ± 7.32	0.001
Protein (%)^ab^	16.41 ± 2.90	17.35 ± 3.12	17.57 ± 2.97	<0.001
Total fat (%)^abc^	38.08 ± 6.11	35.61 ± 5.91	33.58 ± 6.42	<0.001
Alcohol (%)	3.96 ± 3.96	5.05 ± 5.04	4.76 ± 4.62	0.063
Mediterranean Diet Adherence Score^abc^	6.4 ± 1.7	7.8 ± 1.8	8.6 ± 1.8	<0.001

Categorical variables are expressed as n (%) and continuous variables as mean ± standard deviation

p: statistically significant differences (*p* < 0.05)

Post hoc differences: ^a^T1 and T2; ^b^T1 and T3; ^c^T2 and T3

**Table 5:** Energy intake and daily nutrients intake by tertiles of the EVIDENT diet index (Women (*n* = 937))

	TERTILE 1<51.43	TERTILE 2 (51.43 to 53.85)	TERTILE 3 >53.85	
	Mean ± SD	Mean ± SD	Mean ± SD	*p* value
Energy intake(Kcal/day)^c^	2415.0 ± 844.4	2319.5 ± 736.1	2502.9 ± 785.2	0.019
Carbohydrate (g/day)^ac^	257.8 ± 111.0	250.7 ± 94.4	278.1 ± 113.0	0.005
Protein (g/day)^bc^	100.4 ± 31.9	103.0 ± 30.1	111.1 ± 29.5	<0.001
Total fat (g/day)^a^	105.8 ± 39.9	96.6 ± 35.8	101.0 ± 35.8	0.012
Saturated fat (g/day)^ab^	33.9 ± 13.3	29.0 ± 12.4	27.1 ± 10.5	<0.001
Fiber (g/day)^abc^	19.7 ± 9.1	25.9 ± 9.1	33.4 ± 12.5	<0.001
Cholesterol (g/day)^b^	467.5 ± 175.7	449.8 ± 174.8	436.0 ± 162.2	0.080
Carbohydrate (%)^b^	42.15 ± 7.08	42.97 ± 7.18	43.89 ± 7.68	0.016
Protein (%)^ab^	17.07 ± 3.27	18.14 ± 3.19	18.25 ± 3.60	<0.001
Total fat (%)^ab^	39.41 ± 6.39	37.34 ± 6.22	36.32 ± 6.43	<0.001
Alcohol (%)	1.37 ± 2.51	1.55 ± 2.39	1.54 ± 2.82	0.655
Mediterranean Diet Adherence Score ^abc^	6.8 ± 1.9	7.6 ± 1.7	8.6 ± 1.6	<0.001

Categorical variables are expressed as n (%) and continuous variables as mean ± standard deviation

p: statistically significant differences (*p* < 0.05)

Post hoc differences: ^a^T1 and T2; ^b^T1 and T3; ^c^T2 and T3

In a multiple regression analysis, after a complete adjustment, it was estimated that for each one-point increase in the EVIDENT diet index, CVR, SBP, WC, and PWV decreased by 0.14, 0.43, 0.24, and 0.09 respectively (*p* < 0.05 for all). Table 6.

**Table 6:** Multiple regression analysis: Relationship between EVIDENT index with Cardiovascular risk, blood pressure, waist circumference and PWV

	CV Risk			SBP			Waist circumference			PWV^a^		
	β	95% CI	p	β	95% CI	p	β	95% CI	p	β	95% CI	p
Model 1	0.260	0.042 to 0.478	0.020	−0.028	−0.304 to 0.248	0.842	−0.035	−0.108 to 0.038	0.353	−0.029	−0.107 to 0.050	0.476
Model 2	−0.223	−0.377 to −0.069	0.005	−0.444	−0.708 to −0.180	0.001	−0.094	−0.170 to −0.018	0.015	−0.082	−0.147 to −0.017	0.014
Model 3	−0.143	−0.267 to −0.019	0.023	−0.427	−0.685 to −0.168	0.001	−0.241	−0.426 to −0.056	0.011	−0.089	−0.148 to −0.030	0.003

*CV* Cardiovascular risk, *SBP* Systolic blood pressure, *PWV* Pulse wave velocity

p: statistically significant differences (*p* < 0.05)

Model 1: No adjusted

Model 2: Adjusted for age, gender

Model 3: Adjusted for age, gender, smoking, energy intake, antihypertensive, antidiabetic and lipid-lowering drugs

^a^Model 3 (PWV). Adjusted for age, gender, smoking, energy intake, antihypertensive, antidiabetic and lipid-lowering drugs and systolic blood pressure

Independent variable: EVIDENT diet index score

**Fig. 1:**
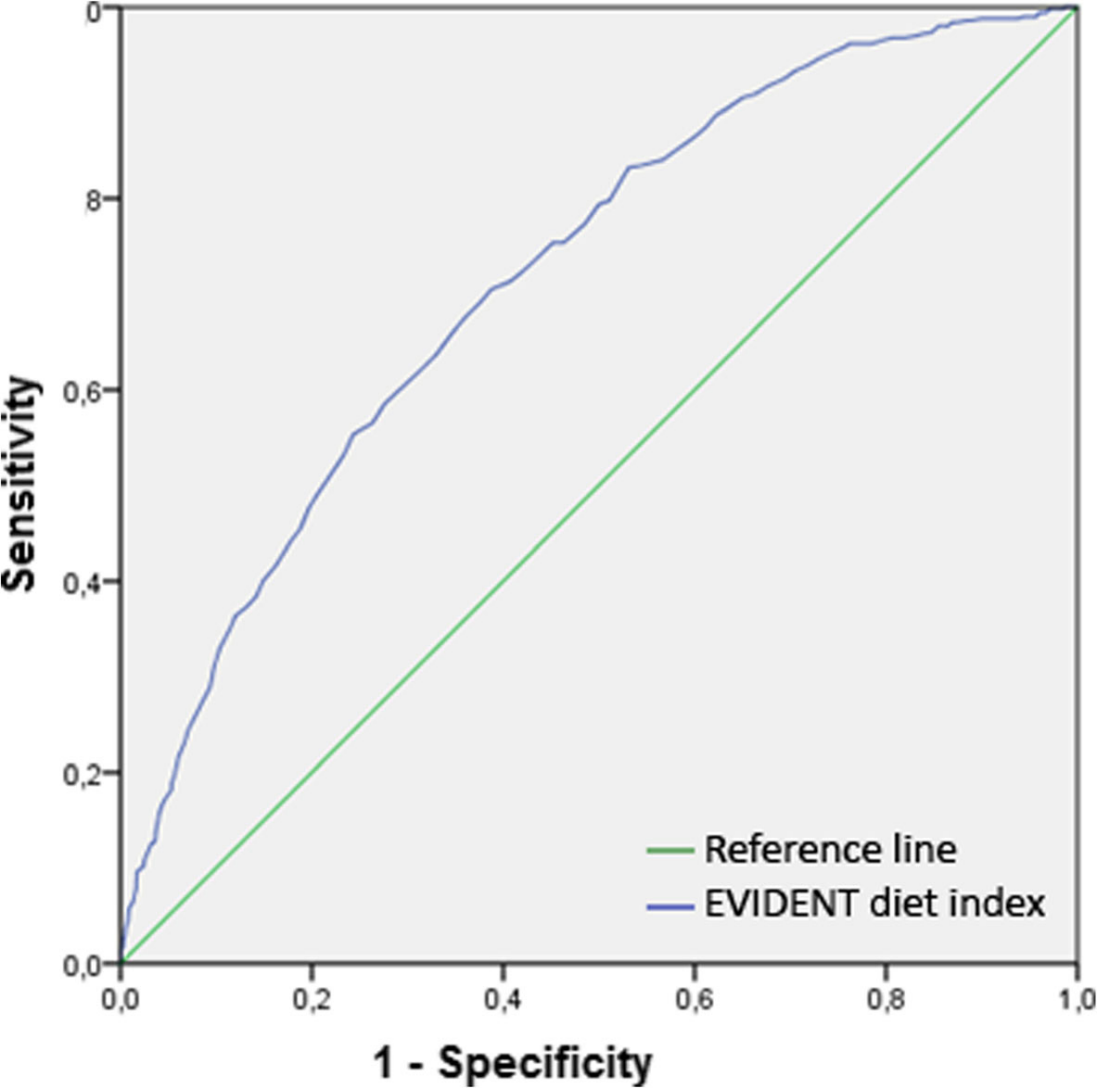
ROC curves for the EVIDENT diet index scores for prediction the adherence to the Mediterranean diet. (Area under the curve 0.72, 95% CI, 0.69–0.75, *p* < 0.001)

In the ROC curve (area under the curve 0.72, 95% CI, 0.69–0.75, *p* < 0.001), the best cut-off point of the EVIDENT diet index for predicting good adherence to the Mediterranean diet was 52.3 (0.71 sensitivity and 0.61 specificity) (Fig. 1).

## Discussion

The EVIDENT diet index (diet quality index derived from a FFQ) shows a close relationship to cardiovascular risk and its components, as well as pulse wave velocity, an intermediate marker in development of atherosclerosis. It has also been shown to be a very important predictor of adherence to Mediterranean diet.

Current research focuses on the study of dietary patterns, rather than analysis of given nutrients [14, 15]. Development of diet quality indices such as the EVIDENT diet index, represents a way to analyze FFQ data from a comprehensive viewpoint, using an approach different from the one most commonly applied to date. As FFQs provide a multitude of data, their routine management is difficult. There has been thus a trend to search for formulas that simplify such information [16]. Today, diet quality indices are mainly used in research, but they could be helpful in primary care clinics because they would allow for systematic, rapid, and simple understanding of the quality of patient diet and its impact on cardiovascular health. This would in turn allow for identifying, selecting, and prioritizing groups of people with poorer quality of diet. From the clinical viewpoint, FFQs allow for estimating mean daily use of energy and nutrients, but development of these quality indices also allows for comprehensive management of all information provided by these FFQs. In addition, as in the case of the EVIDENT diet index, they may be a very significant predictor of adherence to Mediterranean diet. The EVIDENT diet index simplifies the evaluation of diet quality in daily practice. This index is derived directly from the 137-item FFQ. For the daily practice, some foods considered as neutral for the health, It may not be necessary to apply. This would have a direct effect on the evaluation time. In addition, quality indexes allow an overall view of the quality of the diet as a whole, which the FFQ does not provide.

The relationship between cardiovascular risk factors and diet quality was explored in the study conducted by Funtikova et al. [17] with a 10-year follow-up. Our study supports the conclusion that high diet quality is associated to a better profile in cardiovascular risk factors. Several studies have reported an association to blood pressure. One of the most important of these was the DASH study, which concluded that a diet rich in vegetables and fruit and low in saturated fat may decrease blood pressure. [18]. More recently, this relationship was also analyzed in the ENCORE and OMNIHEART studies [19, 20] with the same results. The food groups considered more favorable for cardiovascular health in the EVIDENT diet index included skimmed milk products, vegetables, fruit, as well as other products commonly included in the Mediterranean diet such as olive oil, red wine, legumes, white meat, or fish. This group of foods represents a dietary pattern similar to that of the DASH study but with elements unique to Mediterranean countries. The relationship between diet quality and BMI was previously studied by Asghari et al. [21], who found no significant association. However, Gregory et al. [22] and Sundararajan et al. [23] reported an inverse relationship between diet quality and BMI, which was stronger in females. Our results support this relationship even after including an adjustment for confounding factors such as age, sex, use of drugs, and other lifestyles such as smoking, which did not modify the results.

However, the most important finding may be the association of the EVIDENT diet index to overall cardiovascular risk estimated using the Framingham equation. This finding agrees with the conclusions of a recent study by Sotos-Prieto et al. [24] which suggested a relationship between diet quality and an index encompassing cardiovascular risk factors, as well as an impact on overall mortality figures [25].

Pulse wave velocity is currently considered as the gold standard in evaluation of arterial stiffness [26] and as an intermediate marker in development of cerebrovascular disease. The relationship between heart-healthy lifestyles and pulse wave velocity has been shown in several studies [27, 28]. However, the conclusion reached in the meta-analysis conducted by Petersen et al. [29] was the need for evidence on the relationship between intake patterns and pulse wave velocity, which may be seen in our study. Higher values in the EVIDENT diet index are related to lower pulse wave velocity, even after a complete adjustment model.

Limitations of this study included that cause-and-effect relationships cannot be determined from a cross-sectional study. Future longitudinal studies may help establish causal relationships between this diet quality index and intermediate and final CVD markers. However, the study included a wide adult sample attending health care centers having heterogeneous characteristics and a wide age range, which increases validity of results. Other limitation is referred to the absence of validation and construction of the index that was based in an adapted dietary patterns proposed by other authors adapted to Spanish dietary habits.

## Conclusions

The diet quality index developed from a FFQ was associated to cardiovascular risk and its components, and also to arterial stiffness, as measured with pulse wave velocity. The index is also a good predictor of adherence to Mediterranean diet in the adult population.

## Abbreviations

BMI: Body mass index
CVR: Cardiovascular risk
FFQ: Food frequency questionnaire
MD: Mediterranean diet
PWV: Pulse wave velocity
SBP: Systolic blood pressure
WC: Waist circumference
